# Spiritual Therapy to Improve the Spiritual Well-Being of Iranian Women with Breast Cancer: A Randomized Controlled Trial

**DOI:** 10.1155/2013/353262

**Published:** 2013-08-18

**Authors:** Najmeh Jafari, Ziba Farajzadegan, Ahmadreza Zamani, Fatemeh Bahrami, Hamid Emami, Amir Loghmani, Nooshin Jafari

**Affiliations:** ^1^Community Medicine Department, School of Medicine, Isfahan University of Medical Sciences, Hezar Jerib Street, Isfahan, Iran; ^2^Consultation Department, Psychology school, Isfahan University, Hezar Jerib Street, Isfahan, Iran; ^3^Radiotherapy Department, School of Medicine, Isfahan University of Medical Sciences, Hezar Jerib Street, Isfahan, Iran; ^4^Anesthesiology Department, School of Medicine, Isfahan University of Medical Sciences, Hezar Jerib Street, Isfahan, Iran

## Abstract

*Purpose*. The aim of this study was to investigate the role of spiritual therapy intervention in improving the spiritual well-being and quality of life (QOL) of Iranian women with breast cancer. *Methods*. This randomized controlled clinical trial (RCT) recruited 65 women with breast cancer, randomly assigned to a 6-week spirituality-based intervention (*n* = 34) or control group (*n* = 31). Before and after six-week spiritual therapy intervention, spiritual well-being and quality of life (QOL) were assessed using Functional Assessment of Chronic Illness Therapy Spiritual Well-being scale (FACIT-Sp12) and cancer quality-of-life questionnaire (QLQ-C30), respectively. *t*-test, Paired *t*-test, pearson's correlation, and hierarchical regression analyses were used for analysis using Predictive Analytic software (PASW, version 18) for Windows. *Results*. After six spiritual therapy sessions, the mean spiritual well-being score from 29.76 (SD = 6.63) to 37.24 (SD = 3.52) in the intervention group (*P* < 0.001). There was a significant difference between arms of study (*F* = 22.91, *P* < 0.001). A significant positive correlation was detected between meaning and peace with all subscales of functional subscales on European Organization for Research and Treatment of Cancer quality of Life (EORTC QLQ-C30) (*P* < 0.05). Hierarchical regression analyses of participants indicated that the study arm, pain, and financial impact were significant predictors of spiritual well-being and overall QOL. Social functioning was another significant predictor of spiritual well-being. *Conclusion*. The results of this randomized controlled trial study suggest that participation in spiritual therapy program is associated with improvements in spiritual well-being and QOL. Targeted interventions to acknowledge and incorporate spiritual needs into conventional treatment should be considered in caring of Iranian patients with breast cancer.

## 1. Introduction

Breast cancer is the top cancer among women worldwide with an increasing incidence in developing countries such as Iran [1, 2]. It constitutes 25% of all cancers among Iranian women, with the highest rate in those aged between 35 and 44 years [3, 4].

Diagnosis of breast cancer is a tragic event for a woman [5]. Adjusting to the news of having cancer, informing relatives about disease, planning for treatment and surgery, and treating side effects may cause psychological distress and morbidity in these patients [6]. Several psychosocial interventions have been developed to decline the psychological morbidity associated with the disease and to improve daily functioning and quality of life of these patients. Interventions such as exercise [7, 8], peer support group [9–11], mindfulness-based stress reduction (MBSR) program [12–14], and cognitive behavioral therapy (CBT) [15–17] showed that they can have a positive effect on quality of life of these patients.

Confronting cancer diagnosis and its challenges, many cancer patients seek comfort in spiritual beliefs, which in some cases are associated with positive psychological outcomes [18, 19]. Religious and spiritual coping has been associated with lower levels of distress besides anger, anxiety, and social isolation in cancer patients as well as better adjustment with cancer [20–22]. 

Spirituality is defined as “the aspect of humanity that refers to the way individuals seek and express meaning and purpose, and the way they experience their connectedness to the moment, to self, to others, to nature and to the significant or sacred [23].” Spiritual or religious dimensions are likely to be embedded in issues such as meaning [24, 25], control [26, 27], identity [28–30], and relationships [31, 32]. Interventions that increase spiritual well-being may allow cancer patients to reevaluate life goals, priorities, and sources of meaning in their life and help them to reduce emotional reactivity and increase appreciation for life [33, 34].

Even though research has indicated the significance of spirituality in the quality of life of patients with cancer, there is minimal information in the literature documenting the effect of spirituality-based interventions in different cultures such as Iranian Muslim patients. The results of a recent cross-sectional study showed that Iranian Muslim patients have a lower level of spiritual well-being especially in meaning and peace subscales of Functional Assessment of Chronic Illness Therapy-Spiritual Well-Being (FACIT-Sp12) [35]. This highlights the need to examine the effects of spirituality-based interventions in an Iranian context. Fallah et al. examined the effect of spiritual group intervention on the increase of hope, life satisfaction, and happiness in Iranian women surviving breast cancer and found that this intervention was beneficiary for increasing the mental strengths of these patients [36]. However, there is still lack of evidence regarding results of spiritual therapy in terms of spiritual well-being and its relation to QOL.

 The purpose of the current trial was to study whether a spiritual therapy intervention might be effective in improving the spiritual well-being and quality of life of Iranian women with breast cancer.

## 2. Methods

### 2.1. Design

A randomized controlled clinical trial was undertaken to compare the efficacy of spiritual therapy intervention with standard care in improving the spiritual well-being and QOL of patients with breast cancer undergoing radiation therapy. A detailed description of the methodology has been indicated elsewhere [37].

### 2.2. Participants

Participants were recruited from Breast Cancer Research Center, St. S. Al-shohada hospital, which is the only referral center for cancer treatment and rehabilitation in Isfahan, Iran. Eligibility criteria included age more than 18 years, breast cancer diagnosis within the last 12 months, and a treatment recommendation of radiation therapy of at least 2 weeks. Patients with concomitant chronic disease and major depression disorder and absent in 2 consecutive sessions were excluded. 

Participants were randomly assigned following simple randomization procedures (computerized random numbers) to either the structured intervention arm or standard medical care arm. Participants in control group received standard management and treatment and routine educational program (based on nutrition, physical activity, and radiation therapy patient-education program). Intervention group received routine management/education and also an additional education program based on spiritual therapy intervention.

The major outcomes of this study were spiritual well-being and QOL.

### 2.3. Instruments

To assess the spiritual well-being of participants, we used the Persian version of 12-item Functional Assessment of Chronic Illness Therapy-Spiritual Well-Being (FACIT-Sp12) questionnaire. This is a valid and reliable instrument to provide an inclusive measure of spirituality in research and clinical practice [38, 39]. This questionnaire contains 12 spirituality items and three subdomains of spiritual well-being peace, meaning, and faith (scale range, 0–48; higher scores signifying greater spiritual well-being) [40]. All 3 scales have high internal consistency (Cronbach's alpha for total scale, 0.87; for meaning/peace subscale, 0.81; for faith subscale, 0.88) [38]. This questionnaire is translated and validated in Persian by authors and the psychometric properties of the questionnaire are confirmed [41].

The quality of life in these patients was assessed by European Organization for Research and Treatment of Cancer Quality of Life (EORTC QLQ-C30) [43, 44]. QLQ-C30 incorporates nine multi-item scales: five functional scales (physical, role, cognitive, emotional, and social); three symptom scales (fatigue, pain, and nausea and vomiting); and a global health and quality-of-life scale. Several single-item symptom measures are also included [43].

This questionnaire is translated and validated in Persian by Montazeri et al. in 2000 and they found that the Iranian version of the EORTC QLQ-C30 is a reliable and valid measure of health-related quality of life among women with breast cancer [45].

The questionnaires and demographic data were administered by the researchers in face-to-face interviews.

Sociodemographic data included demographic information (age, marital status, education, occupation, and religion) and concurrent chronic disease, collected through a questionnaire. Clinical data including pathological disease stage was extracted from medical records. 

The intervention was provided over the course of six sessions weekly (Table 1). Each session had a theme incorporating the specific domains of spirituality concluded with a 20- to 30-minute guided relaxation and meditation exercise. Participants received a 50-page manual and a CD-ROM containing written materials and powerpoint slides covered in each of the six sessions for their review. The educators were 3 spiritual healers with great experience in this field. Each of the educators participated in every step of the development of this intervention and used structured, manualized materials. There were back-up persons for each educator who were trained to use the manual and conduct the session.

 Each session lasted approximately 2-3 hours and was balanced with didactic material, a question and answer period, sharing, reflecting, and relaxation and meditation practice. Sessions were recorded as audio and the meditation session was recorded on videotape. These recordings were assessed after each session by independent reviewers to ensure the fidelity of the healers to the protocol. The video-CD of the meditation session was given to the patients as their guidance for home practice. Furthermore, participants were asked to comment on the specific quality of the main theme of each session. This ensured reliability and reproducibility of the interventions over the course of the study.

### 2.4. Analysis

The scale scores of the QLQ-C30 and FACIT-Sp12 were computed as recommended in the scoring manuals [40, 46]. All descriptive statistics are presented as means and standard deviations for quantitative variables and as relative frequencies and percentage for categorical variables. EORTC-QLQ and spirituality scores are presented as means with their 95% confidence intervals. Two-sample *t*-tests were used for the comparison of continuous variables and Pearson's chi-square tests were used for the comparison of categorical variables.

The overall QOL and spiritual well-being before and after study were compared using Paired *t*-test.

Pretherapy versus posttherapy comparisons were carried out using the paired *t*-test if the data was approximately normal or the Wilcoxon signed-rank test if it was not. All comparisons were carried out on a two-tailed basis.

We estimated effect sizes by dividing the mean difference between conditions after intervention by the pooled standard deviation of the groups.

The Pearson correlation analyses were used to study the bivariate relationships between quality of life domains and spiritual well-being.

Hierarchical regression analyses were conducted to examine the study arm as a predictor of spiritual well-being and QOL after controlling for the corresponding baseline variables and to investigate other predictors of these outcomes. 

Data of participants were analyzed by the Predictive Analytic Software (PASW, version 18) for Windows.

### 2.5. Ethics

This trial has been assigned the Iranian Randomized Controlled Trial Registry Number IRCT138904024242N1. The design of the study was approved by Ethics Committee of Vice Chancellor for Research, Isfahan University of Medical Sciences (project number 389319). All participants received trial information and provided written informed consent. Also, the confidentiality of all information was managed carefully by researchers.

## 3. Results

### 3.1. Participant Characteristics

Of the 123 possible participants, 96 patients acquired the inclusion criteria and were enrolled in the study. Sixteen patients were ineligible and excluded due to concurrent chronic disease (*n* = 14) and major depression (*n* = 2). Fifteen patients refused to participate. The most common reasons for refusal, in order of frequency, include lack of interest in research participation (*n* = 7), excessive travel distance to the treatment center (*n* = 5) and not feeling well enough to participate (*n* = 3). In all sixty-five patients (34 patients in the spiritual therapy group and 31 patients in the control group) completed the 6-week intervention and were evaluated for the outcome. 

The average age of the participants in the intervention group and the control group were 47.9 years (SD = 10.56) and 48.1 (SD = 10.2), respectively. Most patients were married (95.3%) and housewives (50.7%). Half of the patients were actively employed. All patients expressed their religious affiliation as Muslims. Of the 65 participants, 62% had mastectomy and 28% had conservative breast surgery. There were no significant differences in distribution of demographic and clinical characteristics between intervention and control groups. Hence, the treatment groups were well balanced at baseline for potentially confounding concomitant variables. 

There were no significant differences between the study arms in these baseline characteristics at the time of randomization. There were no statistically significant differences in demographics between the women who attended and those who dropped out or never attended (*P* > 0.05).

### 3.2. Primary Analyses

Table 2 provides an overview of the baseline and after intervention spiritual well-being scores for the total sample (*n* = 65), including effect sizes.

After six spiritual therapy sessions, the mean spiritual well-being score changed from 29.76 (SD = 6.63) to 37.24 (SD = 3.52) in the intervention group (*P* < 0.001). There was a significant difference between arms of study (*F* = 22.91, *P* < 0.001). There was a significant improvement in all three (meaning, peace, and faith) subscales of FACIT-Sp 12 in spiritual therapy group after intervention (*P* < 0.05). 

All functional scales of EORTC QLQ-C30 were improved after intervention. After six spiritual therapy sessions, the mean global health status score/QOL improved significantly in the intervention group. 

There was no statistically significant difference in QOL and spiritual well-being scores between two times of measurement in the control group (Table 2).

### 3.3. Secondary Analyses

Bivariate relationships were determined between the outcome measures. There was a significant positive correlation between meaning and peace with all subscales of functional subscales on EORTC-QLQ C30 (*P* < 0.05). Faith was significantly correlated with all global quality of life and physical, emotional, and social subscales (Table 3).

As seen in Table 4, hierarchical regression analyses of participants indicated that study arm was a significant predictor of both spiritual well-being and overall QOL. After adjusting with baseline data, pain and financial impact were significant predictors of spiritual well-being and overall QOL. Social functioning was another significant predictor of spiritual well-being.

## 4. Discussion

We have reported the effect of a 6-week spiritual therapy program on the spiritual well-being and QOL of Iranian women with breast cancer. The primary results of this study indicated that the studied population has poor spiritual well-being especially in meaning and peace subscales of FACIT-Sp12. Being Muslim was associated with higher level of faith. Otherwise, our patients reported lower level of meaning and peace. This finding coheres with the results of another study on Iranian Muslim patients [35].

After 6 weeks, intervention participants reported changes in all domains of spiritual well-being and this difference was statistically significant between the intervention and control group. Evidence shows that improvement in spiritual well-being is associated with better adjustment to cancer [47, 48]; hope and positive mood states [49, 50]; functional well-being [51]; reduced hostility, anxiety, and social isolation [52]; and overall well-being and quality of life (QOL) [53–55]. Our analyses confirmed this association and showed a significant influence of the psycho-spiritual intervention on global QOL and physical, role, emotional, cognitive, and social scales of EORTC QLQ-C30. Not surprisingly, our analysis failed to show a significant effect on dyspnea, appetite loss, constipation, and diarrhea symptom scales. This may be due to the more physical (than spiritual) nature of the these symptoms. 

The meaning and purpose in life may help in psychological adjustment following the acute stages of the disease and subsequent treatment [56]. Individuals who experience the existential benefits of this spiritual perspective may also experience better quality of life (QOL), willingness to live [57, 58]; and coping to disease [59]. Kinney et al. showed that an integrated mind-body-spirit self-empowerment program for breast cancer survivors can enable participants to experience a decline in distress, better quality of life, and a deeper sense of meaning and purpose in life as well as a greater sense of wellness [60]. Cunningham described 8-week “Steps Towards Spiritual Healing” program for cancer patients. After trial, the measures for mood, self-efficacy, quality of life, purpose in life, and spirituality demonstrated significant improvements [61]. 

Using the QLQ-C30, significant improvements were shown in the physical well-being scores of fatigue, pain, nausea and vomiting, and sleep disturbance. Previous research has found that activities such as meditation, yoga, and psycho-spiritual therapy can relieve or ease a wide range of physical symptoms [62, 63]. Brady et al. showed that cancer patients who reported a high degree of meaning in their lives were able to tolerate severe physical symptoms greater than patients with lower scores on meaning/peace [39]. 

Meaning and peace subscales of spirituality were moderately correlated with all global general health/QOL, physical, role, emotional, cognitive, and social functioning. There is empirical evidence for the moderate relationship between spirituality and quality of life, supporting the theoretical framework that spirituality is seen as a unique concept that stands in relationship to quality of life [64]. The existential meaning and peace components of spirituality were more strongly related to psychological adjustment than were faith. This result is in line with a large Australian study on 449 cancer patients, indicated that spiritual well-being has a positive association with health-related QOL domains, while the meaning/peace component is more highly related to QOL than the faith component [65]. 

In the present study, we used hierarchical multiple regression analysis to examine the association of spiritual well-being with QOL in survivors of breast cancer. Our results suggest that being in the spiritual therapy group was significantly associated with better spiritual well-being after controlling for disease and demographic variables. Pain, social functioning, and financial impact can predict the spiritual well-being of breast cancer survivors. This is in line with the results of a recent structural equation modeling of FACIT Sp12 while showed that spiritual well-being is positively associated with religiosity, self-esteem, and social relatedness, and is negatively associated with physical suffering [66]. 

On the other hand, pain and financial impact were significant predictors of global health/QOL. Cancer pain significantly affects quality of life and survival of patients with cancer [67]. Pain is present in 14%–100% of cancer patients and is associated with depression and has the most disruptive influence on the quality of life of cancer patients [68]. In addition, cancer treatment has a serious impact on financial aspects of patients' lives and seems to be associated with a poor quality of life [69]. This obviates a multidisciplinary approach in cancer treatment to comply with the needs of survivors.

An important aspect of the current study refers to the inclusion of spiritual therapy as an effective intervention for patients' spiritual well-being and QOL in a religious context. Iran is a religious country and 98% of its population are Muslims [70]. Qualitative studies from Iran showed that spiritual approach is the major coping strategy to respond to cancer and Iranian cancer patients consider spirituality as a source of hope [18, 71–73]. The faith component of spirituality is most often associated with religion and religious belief, whereas the meaning component of spirituality appears to be a more universal concept [74]. This study provides evidence of the effectiveness of spiritual therapy in terms of meaning and peace subscales of spiritual well-being in an Iranian context.

Our study, while having much strength, involved some limitations that should be considered. This study had a small sample size, which reduced the statistical power. An additional limitation concerns the fact that the beneficial psychosocial effects of our study may be due to the positive effects of peer support in these patients. The lack of an “attention control group” in our study does not allow us to attribute all positive outcomes to spiritual therapy intervention. Furthermore, there was no follow-up program after six weeks to assess the effects of intervention. Future research with larger sample size should examine the effect of spirituality-based intervention on other types of cancer or patients with different religious beliefs. 

## 5. Conclusion

The results of this randomized controlled trial study suggest that participation in spiritual therapy program is associated with improvements across spiritual well-being and several areas of quality of life, including physical, emotional, and social functioning. Targeted interventions to acknowledge and incorporate spiritual needs into conventional treatment, should be considered in caring of Iranian patients with breast cancer.

## Figures and Tables

**Table 1:** Spiritual therapy intervention.

Sessions	Main theme	Definition
Session 1	Introduction	Defining the course and introduction. In this session, the participants discussed the possibility of finding or creating meaning out of their experience of cancer. We asked patients to recognize the inner conflict and punishing feelings they have towards themselves and to facilitate a more positive meanings of their cancer experience

Session 2	Relaxation and meditation	Teaching relaxation and meditation by a qualified mentor. All sessions included instruction and active meditation practice. The patients were encouraged to practice this technique individually at home twice a day and given a video-compact disk for guidance

Session 3	Control	This session focused on two aspects of control: things under personal control and things beyond the personal control. We assisted participants to differentiate between these two and write their concerns in two different circles. By focusing on the circle labeled “under God's control,” participants were asked to concentrate on the visualized God's presence around them as a white light and put uncontrolled concerns and problems under God's control. For things under their personal control, we invited participants to use a collaborative approach and view God as a supportive, kind, and helpful partner toward conflict resolution

Session 4	Identity	In this session participants were encouraged to express their grief associated with their disease. We asked them to explore the negative and positive feelings and affirm their strengths and positive attributes inside themselves connecting with them to fight against cancer. Imagining God's presence as a witness to their loss and pain helped participants feel that their losses are acknowledged and guided them to accept and affirm the individual's self-worth

Session 5	Relationships	The focus of this session was on three types of relationships: relationships with oneself, with others, and with God. Listening to one's feelings, positive self-talk, and self-care helped patients to facilitate the relationship with oneself. To resolve any negative feeling about relationship with others, a version of the ‘‘two chair” technique—employed by Gestalt psychologists—was used [42]. Participants were encouraged to concentrate on their relationship with God and any emotion such as guilt, anger, or neglect that they may feel toward God. Then, patients led through ‘‘Circle of Light” guided imagery and talked to God closely

Session 6	Prayer therapy	Encouraging the participants to pray and talk to God closely based on their religious and spiritual believes and ask Him to help them in this process

**Table 2:** Mean baseline and posttrial spiritual well-being score by group (*n* = 65).

Measure	Intervention group *n* = 34				Control group *n* = 31				Effect size	*P* value
	Baseline		After trial		Baseline		After trial	
	Mean	SD	Mean	SD	Mean	SD	Mean	SD
FACIT-Sp12 Scores										
Meaning	10.21	2.96	12.09	1.50	9.45	3.15	9.55	3.14	0.33	<0.001
Peace	7.97	2.44	11.41	1.46	7.41	3.09	7.54	2.46	0.74	<0.001
Faith	11.59	2.90	13.74	1.75	11.06	3.08	11.45	2.33	0.31	<0.001
Total	**29.76**	**6.63**	**37.24**	**3.52**	**27.93**	**7.11**	**28.55**	**5.32**	**0.61**	**<0.001**
QLQ C-30 functional scales										
Global QOL/general health	44.37	13.03	68.63	10.86	37.90	22.44	39.25	15.98	0.78	<0.001
Physical functioning	71.76	12.71	63.60	19.53	62.58	22.15	61.94	19.79	0.44	0.02
Role functioning	61.11	25.82	76.96	20.10	67.20	25.63	61.83	26.94	0.49	0.01
Emotional functioning	44.14	20.49	65.44	13.31	42.47	23.70	36.56	21.80	0.67	<0.001
Cognitive functioning	53.15	25.10	68.14	17.09	55.91	25.29	53.23	24.12	0.49	0.005
Social functioning	49.10	27.20	71.08	19.80	45.70	28.20	42.47	26.11	0.63	<0.001
QLQ C-30 symptom scales/items										
Fatigue	59.46	19.28	37.58	17.51	53.05	24.79	60.57	23.10	0.61	<0.001
Nausea and vomiting	25.68	27.10	18.63	18.24	26.88	24.97	23.12	19.08	0.53	0.03
Pain	45.95	22.36	29.90	16.80	45.16	23.25	48.92	21.05	0.62	<0.001
Dyspnea	19.82	29.87	18.63	18.69	29.03	31.90	33.33	24.34	0.10	0.83
Sleep disturbance	45.95	31.77	36.27	26.42	47.31	35.25	54.84	39.01	0.43	0.03
Appetite loss	37.84	27.40	30.39	25.11	33.33	28.54	38.71	35.58	0.02	0.20
Constipation	32.43	33.78	32.35	30.13	24.73	35.45	34.41	34.94	0.01	1.00
Diarrhea	14.41	25.50	13.73	26.10	17.20	24.14	15.05	24.09	0.01	0.42
Financial impact	66.67	34.24	43.14	29.04	66.67	28.54	68.82	30.95	0.39	<0.001

**Table 3:** Pearson's correlation (*r*-values) between spiritual well-being (FACIT-Sp12) and functional subscales on EORTC-QoL C30.

	Meaning	Peace	Faith
Global QOL/general health	0.518**	0.732**	0.334**
Physical functioning	0.364**	0.445**	0.385**
Role functioning	0.409**	0.379**	0.105
Emotional functioning	0.529**	0.701**	0.286*
Cognitive functioning	0.246*	0.411**	0.047
Social functioning	0.483**	0.653**	0.393**

**Correlation is significant at the 0.01 level (2-tailed).

*Correlation is significant at the 0.05 level (2-tailed).

**Table 4:** Significant predictors of spiritual well-being and overall global quality of life.

Dependent variable	Model	Standardized beta	*P* value	Standard error of the estimate	*R* square
Spiritual well-being	Group	−0.499	0.000	1.073	0.76
	Pain score	−0.302	0.000	0.025	
	Social functioning	0.273	0.003	0.021	
	Financial impact	0.196	0.013	0.016	

Global QOL	Group	−0.505	0.000	2.904	0.78
	Pain score	−0.295	0.000	0.072	
	Financial impact	−0.165	0.022	0.043	
